# Exploring the Potential of Carbon Dots to Combat COVID-19

**DOI:** 10.3389/fmolb.2020.616575

**Published:** 2020-12-17

**Authors:** Sabna Kotta, Hibah Mubarak Aldawsari, Shaimaa M. Badr-Eldin, Nabil A. Alhakamy, Shadab Md, Anroop B. Nair, Pran Kishore Deb

**Affiliations:** ^1^Department of Pharmaceutics, Faculty of Pharmacy, King Abdulaziz University, Jeddah, Saudi Arabia; ^2^Department of Pharmaceutics and Industrial Pharmacy, Cairo University, Giza, Egypt; ^3^Center of Excellence for Drug Research and Pharmaceutical Industries, King Abdulaziz University, Jeddah, Saudi Arabia; ^4^Department of Pharmaceutics, King Abdulaziz University, Jeddah, Saudi Arabia; ^5^Department of Pharmaceutical Sciences, College of Clinical Pharmacy, King Faisal University, Al-Ahsa, Saudi Arabia; ^6^Department of Pharmaceutical Sciences, Faculty of Pharmacy, Philadelphia University, Amman, Jordan

**Keywords:** antiviral, carbon dots, COVID-19, SARS-CoV-2, functionalization of carbon dots

## Abstract

Viral diseases are considered as a global burden. The eradication of viral diseases is always a challenging task in medical research due to the high infectivity and mutation capability of the virus. The ongoing COVID-19 pandemic is still not under control even after several months of the first reported case and global spread. Neither a specific drug nor a vaccine is available for public use yet. In the pursuit of a promising strategy, carbon dots could be considered as potential nanostructure against this viral pandemic. This review explores the possibility of carbon nano-dots to combat COVID-19 based on some reported studies. Carbon dots are photoluminescent carbon nanoparticles, smaller than 10 nm in dimension with a very attractive photostable and biocompatible properties which can be surfaced modified or functionalized. These photoluminescent tiny particles have captured much attention owing to their functionalization property and biocompatibility. In response to this pandemic outbreak, this review attempts to summarize the potential use of carbon dots in antiviral therapy with particular emphasis on their probable role in the battlefront against COVID-19 including their possible biosensing applications.

**Graphical Abstract:**
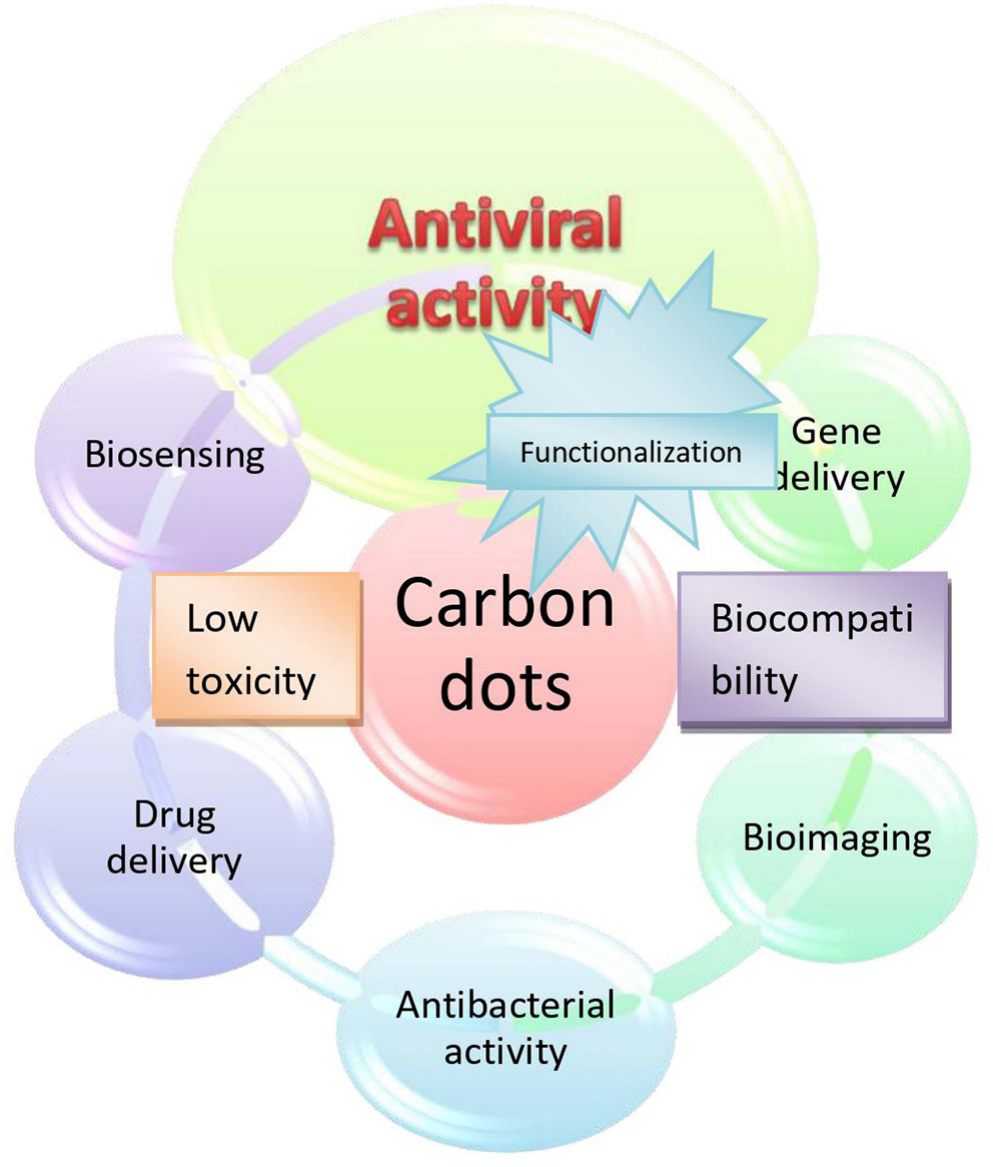


## Introduction

Finding a solution by any means to stop the COVID-19 outbreak in this urgent scenario would be an applaudable step. The global scientific community is struggling to develop sprint technologies to fight this pandemic (Rai et al., 2020). Because of their exceptional properties and biocompatibility, carbon dots can be investigated as a promising solution in the pursuit of an effective therapeutic strategy against COVID-19 (Garg et al., 2020). Exploring these nanostructures for specific and tailored functions is in need to provide solutions for exigencies like COVID-19. Carbon dots have shown proven antiviral effects and that too against Coronaviruses (Du et al., 2018; Ting et al., 2018). At present environment-friendly carbon dots are available for various applications (Du et al., 2014; Park et al., 2014; Yuan et al., 2016). The existence of nanosized functional substances in natural systems has captured a great curiosity for the scientific community due to their unique properties.

Every year millions of people are killed by viral infections and nearly one-third of global mortality is due to infectious diseases. And now the new emerging viral diseases like COVID-19, SARS, MERS, etc. add on to this. Even though vaccination is the best approach to prevent this pandemic situation, unfortunately, it is not yet practical for newly emerging infections. Nanotechnology has tremendous potential in different areas to fight against COVID-19, which include diagnosis, prevention as well as treatment. The application of nanotechnology in antiviral therapy is still in its early stages (Borah et al., 2020; Chen and Liang, 2020; Innocenzi and Stagi, 2020; Palmieri and Papi, 2020). Constant emergences of novel viruses are challenging and need more attention in research on nanotechnology-based targeted antiviral therapy. In this review, we are focusing on the potential therapeutic applications of carbon dots against COVID-19. Once infected with SARS-CoV-2 the patients may need treatment to stop the replication of the virus inside the body. The mechanism of SARS-CoV-2 infection has been already reported in the literature (Coperchini et al., 2020; Dhama et al., 2020; Li et al., 2020; Wang et al., 2020). Reports suggest that the SARS-CoV-2 virus binds to the angiotensin converting enzyme 2 (ACE2) receptor with its spike protein and the virus is around 60–140 nm in size (Chan, 2020). These two findings can be exploited for the design and development of potential tools for the treatment based on nanotechnology. There are several studies done on viral research based on nanotechnology which proves the powerful ability of nanoparticles as antiviral agents. Moreover, functionalized nanoparticles have been proved for its extremely powerful inhibition on proliferation of viruses (Chen and Liang, 2020). Out of these, carbon dots are gaining special interest owing to their exceptional cell membrane permeability, biocompatibility, low cytotoxicity, and functionalization property (Wang and Hu, 2014; Georgakilas et al., 2015; Lim et al., 2015). Carbon dots or carbon quantum dots are now an emerging group of carbon nanoparticles with <10 nm in size and luminescent property (Molaei, 2019). Xu et al. found this luminescent carbon for the first time in 2004 while purifying single-walled carbon nanotubes (Xu et al., 2004). Since carbon dots have several desirable properties like low cytotoxicity, biocompatibility, inertness, photostability, easier synthesis, and functionalization, etc. these are gaining more research interests since its discovery (Zuo et al., 2016; Al-Qattan et al., 2018; Mahajan et al., 2018; Maheshwari et al., 2019; Tian et al., 2020). Apart from drug delivery or therapeutic applications they are well-established for *in vitro* as well as *in vivo* bio-imaging, chemiluminescence, optical sensing, photocatalysis, etc. They are also well-known for their low or no cell toxicity and biocompatibility. Zebrafish larvae showed a normal growth after treating with 1.5 mg/mL carbon dot solution (Kang et al., 2015). Moreover, HeLa cell viability (more than 90%) was observed after incubation of 24 h with 500 μg/mL of carbon dots and the observed LC_50_ was above 5 mg/mL (Ding et al., 2013). Several researches have proved the non-toxicity and safety of carbon dots for *in vivo* applications in animal models. Neutral carbon dots are more promising for biological applications since they do not induce any cellular abnormalities. Negatively charged carbon dots may induce oxidative stress and positively charged carbon dots may be cytotoxic also (Wang K. et al., 2013; Havrdova et al., 2016; Emam et al., 2017). Poor stability and difficult to maintain properties for longer periods of time are another limitations of these nanosized carbon particles (Mishra et al., 2018). The regulatory concerns are similar to nanoparticles. To focus on the regulatory issues on nanoparticles, European Medical association have already created an expert group on nanomedicines. A new concept, Safe-by-Design concepts can be used to anticipate the risk identification, reduction and ambiguities regarding human health and environmental safety in early stages of nanotechnology related product development (Schmutz et al., 2020).

Extensive research have been done by many scientists to prove the ability carbon dots in photodynamic therapy, cancer therapy, antimicrobial therapy etc. (Hola et al., 2014; LináChee and JunáLoh, 2015; Bing et al., 2016; He et al., 2018; Li et al., 2018). A recent review by Basak et al., also gives the potential of carbon dots against viral infections briefly along with some other potential nanomaterials (Basak and Packirisamy, 2020). A large number of reviews are published recently mentioning the potential and possibilities of nanomaterials to fight against corona virus (Innocenzi and Stagi, 2020; Manivannan and Ponnuchamy, 2020; Mukherjee et al., 2020; Nair et al., 2020). But our review focus on the possibility of carbon dots and also functionalized or doped carbon dots against viral infection with special emphasis to corona virus.

Different approaches have been developed for the production of carbon dots, which are mainly categorized as top-down and bottom-up approaches. Some of the most widely used methods include hydrothermal synthesis, pyrolysis, microwave-assisted synthesis, electrochemical oxidation, laser-ablation, etc. from a different diversity of carbon sources (Lim et al., 2015). Some of the precursor molecules for bottom-up approach include ethylene glycol (Hu et al., 2013), boric acid/glycine (Jahan et al., 2013), ethylenediamine and citric acid (Fahmi et al., 2016; Łoczechin et al., 2019) and various green sources like apple juice (Mehta et al., 2015), bee pollen (Zhang et al., 2015), cabbage (Alam et al., 2015), carica papaya juice (Kasibabu et al., 2015), garlic (Zhao S. et al., 2015), ginger (Li et al., 2014), Grape peel (Xu et al., 2015), grass (Liu et al., 2012), honey (Yang et al., 2014), milk (Wang and Zhou, 2014), etc., whereas candle soot (Liu et al., 2007), lampblack (Wang X. et al., 2013), graphite (Anilkumar et al., 2011), etc. are examples of some of the precursor compounds for top-up approach. After synthesis or during synthesis, modification can be achieved by techniques like surface passivation (Zhu et al., 2009; Lai et al., 2012), inorganic salt doping (Anilkumar et al., 2011) and element doping (Dong et al., 2012; Jiang et al., 2012). Surface passivation and functionalization further expand their exploitation from biosensing to drug delivery. For green carbon dots, doping or excessive surface passivation is not necessary since these are almost self-passivated during the nucleation process (Miao et al., 2016).

This review article is exploring the potentials and possibilities of carbon dots against SARS-CoV-2 based on the published research data on antiviral activity of carbon dots. There are not much research done on the applicability of carbon dots against corona virus especially SARS-CoV-2 virus. But the article details all the possible mechanisms by which carbon dots can act against virus and especially corona virus. This review is not detailing the properties, synthesis, and other applications of carbon dots since these are extensively discussed in many reviews (Baker and Baker, 2010; Zhao A. et al., 2015; Yuan et al., 2016; Jaleel and Pramod, 2018). We anticipate that this review article would offer precious insight and cheer up the scientific community for a deeper exploration of therapeutic and diagnostic applications of carbon dots against SARS-CoV-2.

## Antiviral Mechanism of Action of Carbon Nano-Dots

Carbon nano-dots act by a different mechanism at different stages of viral replication. The mechanism of viral infection generally involves four main steps namely attachment, penetration, replication, and finally budding.

### Viral Inhibition by the Alteration of Attachment and Penetration Step

**Figure 1:**
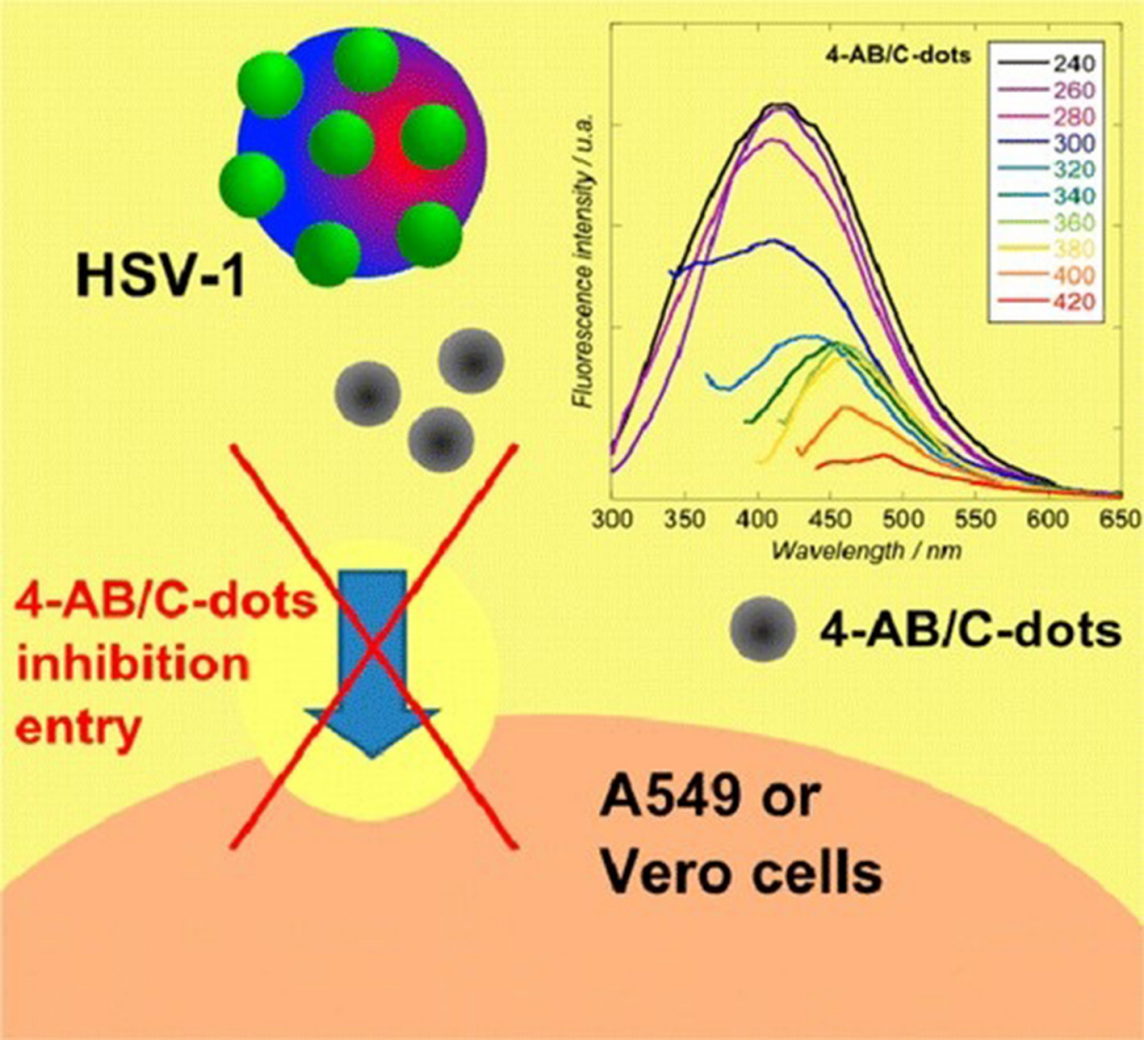
Mechanism of inhibition of entry step by 4-aminophenylboronic acid hydrochloride derived carbon dots. Reprinted with permission from Barras et al. (2016). Copyright (2016) American Chemical Society.

Viral attachment to the host cell is the first step of infection, thus hindrance to this step will inactivate the virus. Most of the reported carbon dots act by interfering with the early stage of viral infection by altering the viral surface proteins. Benzoxazine monomer derived carbon dots can inhibit host-cell entry of Japanese encephalitis virus and other flaviviruses. Immunofluorescence assay in Vero cells showed that these carbon dots can significantly inhibit Zika and dengue virus proliferation. The *in vitro* assay showed that the inhibitory effect on infection is due to the direct contact of carbon dots with the virus membrane, not because of the host cells mounting an antiviral reaction. It was found that the viral binding with the host cell was significantly decreased by the treatment of the Japanese encephalitis virus with Benzoxazine monomer derived carbon dots (Figure 1) (Barras et al., 2016) shows the mechanism of inhibition of entry step by 4-aminophenylboronic acid hydrochloride derived carbon dots.

Inhibition of penetration and viral entry can be brought about by altering the cell surface membrane and attached proteins. The plaque reduction analysis showed a strong concentration related inhibitory action of carbon dots from curcumin on the porcine epidemic diarrhea virus. The curcumin derived carbon dots can block the infection in a very early stage of viral entry. Raman spectral analysis and fluorescence analysis verified that viral aggregation and inactivation is caused by electrostatic interaction of positively charged carbon dots (Ting et al., 2018). Surface-functionalized carbon dots with amine or boronic acid functional groups can obstruct the entrance of type 1 herpes simplex virus. This can in particular act on the very initial step of the viral entry by interacting with the virus or possibly with the cells simultaneously (Barras et al., 2016).

### Viral Inhibition by Inhibiting Replication

Once the virus enters the host cell, the only strategy for inhibition is either to stop the replication or to stop or prevent budding. Inhibition of viral replication can be accomplished by the alteration of enzymes that are needed for viral genome replication. Curcumin carbon dots can drastically slow down the production of negative RNA strand in porcine epidemic diarrhea virus, proved by the reduction in the level of negative-strand RNA in curcumin carbon dots treated cells as compared with the untreated plate at various time intervals after infection. The replication of porcine epidemic diarrhea virus in Vero cells showed decreased plaque numbers as well as reduced virus titers in the carbon dot-treated group as compared to the control group (Ting et al., 2018).

### Viral Inhibition by Hindering Budding and Detachment Steps

After replication, the progeny will bud-off from the host cell as a new virus. The strategies which can prevent the budding and excision of newly formed more virulent virus can also inhibit or control the infection.

Some viral infections are characterized by overexpression of reactive oxygen species which in turn leads to DNA damage through apoptotic regulation signaling pathways. Curcumin derived carbon dots can inhibit reactive oxygen species (ROS) generation which is induced by coronavirus infection (Ting et al., 2018).

Even though these three are the major antiviral mechanisms of action carbon dots against virus, many research have proved the antiviral activity without mentioning exact mode of action. An elaborated research is needed to explore all the possible mode of operation of carbon dots in inactivating or suppressing or killing of virus.

## Antiviral Carbon Dots

In a recent article by Garg et al., elaborated the inhibitory mechanism of human coronaviruses by hetero atom doped carbon dots. The research group propose the potential development of triazole-based carbon dots against SARS-CoV-2 infection using a series of bioisosteres. Since carbon dots have a large number of hydrophilic functional groups on borders, they are appropriate for diverse biomedical applications. In addition to this the surface functionality of these magic nano substance is vital to fine-tune the of interaction level with virus (Garg et al., 2020).

**Figure 2:**
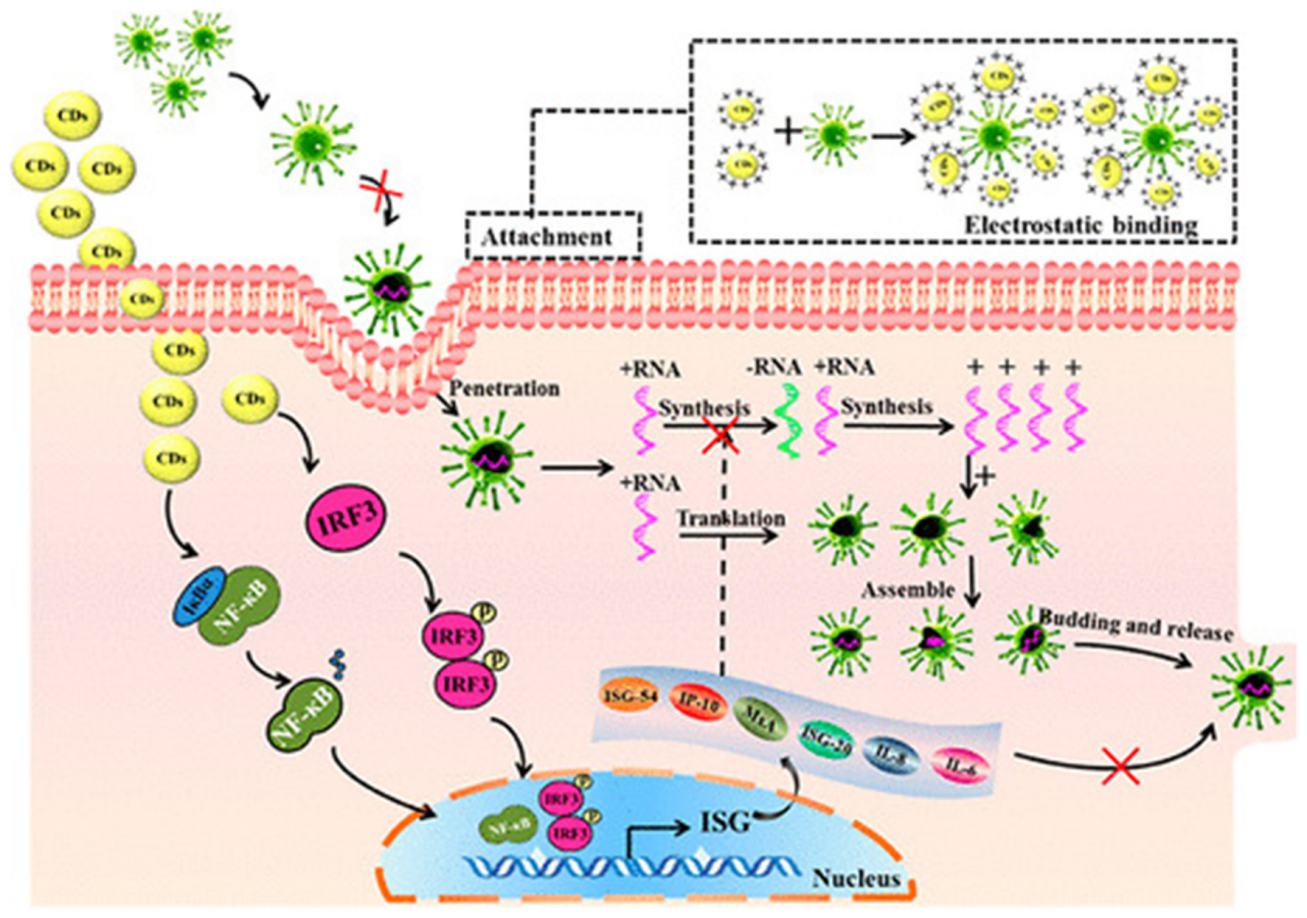
Mechanism of viral inhibition of cationic curcumin carbon dots. Reprinted with permission from Ting et al. (2018). Copyright (2018) American Chemical Society.

Curcumin cationic carbon dots (CCM-CDs) can efficiently inhibit coronavirus infection. Curcumin carbon dots were synthesized by the hydrothermal reaction of curcumin and citric acid in a Teflon coated autoclave followed by purification with centrifugation and then dialysis. The CCM-CDs were found to inhibit the entrance of virus, production of the negative strand of RNA as well as budding. Suppression of viral replication was found to be due to stimulation in the production of interferon stimulating genes as well as pro-inflammatory cytokines and also due to the accumulation of ROS. This was proved as a multisite inhibitor for Enteric Coronavirus (Figure 2). This one step ultrasmall sized (1.5 nm) antiviral fluorescent CCM-CDs with a positive charge and many hydrophilic groups obtained by pyrolysis of curcumin are highly effective against coronavirus model (porcine epidemic diarrhea virus) (Ting et al., 2018).

Carbon dots can effectively inhibit the replication of RNA viruses like Porcine reproductive and respiratory syndrome viruses. Carbon dots are synthesized by the hydrothermal reaction of PEG-diamine and ascorbic acid in a Teflon coated autoclave chamber. The antiviral activity was tested *in vitro* on Monkey kidney cells infected with Porcine reproductive and respiratory syndrome viral strain, WUH3. Viral replication is inhibited by increased interferon-α production and enhanced expression of interferon-stimulating genes (Du et al., 2016). A broad strategy of anti-coronavirus therapy is not practically possible due to the biodiversity and rapid mutation characteristic of coronaviruses. Loczechin et al. developed seven different types of carbon quantum dots against human coronavirus. The first generation carbon dots were made from ethylenediamine/citric acid by hydrothermal carbonization and then functionalization was carried out by chemical integration of boronic acid. The second-generation carbon dots were prepared from 4-aminophenyl boronic acid. Inhibition of HCoV-229E entry as well as viral replication was achieved with the developed carbon dots (Łoczechin et al., 2019). Boronic acid or amine group surface functionalized carbon dots can inhibit type 1 herpes simplex virus infections. The carbon dots were synthesized from 4-aminophenyl boronic acid hydrochloride by hydrothermal carbonization showed a high potency to prevent the infection in herpes simplex type 1 infected A549 and Vero cells. The research showed that the carbon dots interfere with the entry of the virus into the host cell (Barras et al., 2016). Carbon dots derived from benzoxazine monomers by hydrothermal reaction was found to be effective against the adenovirus-associated virus, porcine parvovirus, dengue virus, Zika virus, and Japanese encephalitis virus. Carbon dots were formed as a result of pyrolysis, carbonization, and oxidization of benzoxazine monomers in the presence of aqueous sodium hydroxide (NaOH) in a Teflon coated stainless steel autoclave. These carbon dots were able to bind directly to viral surface proteins and stop the first step of vial attachment with the host cells (Huang et al., 2019).

Curcumin derived carbon quantum dots were found to be effective against enterovirus 71 also. One-step heating at a temperature of 180°C preserved polymeric curcumin moieties with advanced antiviral properties. The core-shell of carbon dots is formed by dehydration, polymerization, carbonization, and surface passivation of curcumin with pyrolytic curcumin like polymer surface, polymerization, crosslinking carbonization, and surface passivation. Insoluble black carbon materials are formed as a result of severe pyrolysis as well as carbonization of curcumin at higher temperatures. In this reaction, curcumin acts both as a source of carbon and also as a source of surface functionalization moiety. In new born mice infected with a lethal dose of enterovirus 71, this curcumin derived, biocompatible carbon dots was able to decrease mortality and protects from virus-induced paralysis of the hind limb. The antiviral effect is due to the inhibition of viral attachment, promotion of antioxidant action, and also the alteration of transcription regulation, activation of intracellular signaling cascades (Lin et al., 2019). Surface modified carbon dots were able to produce a significant antiviral effect on human norovirus virus-like-particles (VLP) by inhibiting binding to histo-blood group antigen receptors on human cells. In this work, chemical functionalization was carried out on harvested carbon nanoparticles from the commercially available carbon nano powders. The 2,2′-(ethylenedioxy)-bis(ethylamine) (EDA) functionalization was achieved by refluxing carbon nanoparticles with thionyl chloride and mixing with dried EDA liquid under heat and nitrogen environment. EDA carbon dots were separated after centrifugation and dialysis against water. It was found that the carbon dots were able to inhibit the binding of human norovirus virus-like particles to saliva A, B, and O type HBGA receptors. It could also inhibit VLPs' binding to their corresponding antibodies. The study could prove the antiviral property of carbon dots by inhibiting the binding of viruses with HGBA receptors. The paper points out that this strategy could be effectively used in preventing or spreading of human Norovirus infection since there is no effective vaccine. The strategy of disabling viral recognition of binding sites on host cells can be used as a promising antiviral approach (Dong et al., 2017).

Highly biocompatible carbon dots from glycyrrhizic acid were able to inhibit the entry as well as reproduction of porcine reproductive and respiratory syndrome virus. These carbon dots were produced by hydrothermal process by the addition of NaOH in a Teflon-lined autoclave. These carbon dots possess the ability to inhibit the accumulation of ROS in the cell as well to stimulate the innate immune response. Moreover, it could inhibit porcine epidemic diarrhea virus and pseudorabies virus which suggests its broad spectrum of antiviral activity (Tong et al., 2020).

Du et al., developed Glutathione capped CdTe quantum dots against pseudorabies virus. From the growth curve as well as fluorescence co-localization analyses it was clear that CdTe QDs inhibit viral multiplication at an early stage by controlling the invasion, and also found that it has no significant action against viral penetration. The size of quantum dots decreased gradually by addition of virus within 30 min due to the of release Cd^2+^ by interaction with the virus and as result a reduction in the number virus which can infect cells was achieved. The structure of viral surface proteins is also altered was evidenced by Raman spectra and Circular Dichroism spectroscopy analyses. This research gives an in-depth understanding of the inhibition of viral pathogenesis by carbon dots (Du et al., 2015).

Liu et al. developed two types of carbon dots by a hydrothermal process which can selectively enter into the cytoplasm and the whole cell with no surface modification. Blue-fluorescent carbon dots were prepared by heating powdered young barley leaves with anhydrous citric acid in a stainless steel autoclave. Cyan-fluorescent carbon dots were produced in a similar way as the previous one with the addition of urea along with citric acid. The carbon dot with blue fluorescence can only enter the cytoplasm but with comparatively better antiviral property against pseudorabies virus, while the cyan-fluorescent carbon dot was cable of distribution over the entire cell, including the nucleus (Liu et al., 2017).

Quantum dots prepared by using microwave were able to inhibit viral replication both *in vivo* and *in vitro* by the inhibition of cellular nuclear factor κB signaling pathway. This pathway has a vital role in the inflammatory response. The carbon dots were prepared similar to their previous work by microwave synthesis of aqueous dispersed CdTe/CdS/ZnS Core-Shell-Shell procedure which produced biocompatible and photostable carbon dots. Apart from antiviral activity, the carbon dots exhibited anticancer and anti-inflammatory activity (Hu et al., 2016).

Carbon dots can successfully prevent HIV 1 infection through inhibition of target cell interaction by interfering with the entry step. Carbon dots are synthesized by citric acid pyrolysis and functionalized with boronic acid since it ensures the specific interaction of carbon dots with glycoprotein on the viral surface. This boronic acid conjugated carbon dots was able to bind to gp120 protein on the virus and stop the binding of MOLT-4 cells and block infection. *In vitro* experiments proved that higher concentrations of boronic acid conjugated carbon dots on syncytia, which mediate fusion of infected cells with an adjacent cell, were observed in the cultured cells. The absence of cellular toxicity is proved on MOLT-4 human leukemia cells by specific assays. The results offer a basis for the advanced exploration of functionalized carbon dots in antiviral therapy (Fahmi et al., 2016). The published researches on carbon dots for the antiviral effect are summarized in the Table 1.

**Table 1:** Antiviral carbon dots.

**Carbon dot (size)**	**Synthesis method /precursor**	**Effective against**	**Mechanism of viral inhibition**	**References**
Curcumin cationic carbon dots (1.5 nm)	Hydrothermal reaction/ curcumin and citric acid	Coronavirus model (porcine epidemic diarrhea virus)	Entry, replication, and budding	Ting et al., 2018
Carbon dots (4.7 nm)	Hydrothermal reaction /PEG-diamine and ascorbic acid	Porcine reproductive and respiratory syndrome virus	Replication	Du et al., 2016
Functionalized carbon quantum dots	Hydrothermal carbonization/ethylenediamine and citric acid	Human coronavirus	Entry and replication	Łoczechin et al., 2019
Boronic acid/amine-functionalized carbon dots	Hydrothermal carbonization/4-aminophenyl boronic acid hydrochloride	Herpes simplex virus type 1	Entry	Barras et al., 2016
Benzoxamine carbon dots (4.4 nm)	Hydrothermal reaction/benzoxazine monomers	Adenovirus-associated virus, Porcine parvovirus, Dengue virus, Zika virus, and Japanese encephalitis virus	Attachment	Huang et al., 2019
curcumin derived carbon quantum dots (4.2–5.2 nm)	Pyrolysis/curcumin	Enterovirus	Entry and replication	Lin et al., 2019
Carbon dots	2,2′-(ethylenedioxy)bis(ethylamine) and 3-ethoxypropylamine	Human norovirus virus-like-particles	Inhibition of binding	Dong et al., 2017
Glycyrrhizic acid carbon dots (11.4 nm)	Hydrothermal/Glycyrrhizic acid	Coronavirus and Herpes viridae (porcine reproductive and respiratory syndrome virus)	Invasion and Replication	Tong et al., 2020
Blue-fluorescent carbon dots (1.9 nm) Cyan-fluorescent carbon dots (2.7 nm)	Hydrothermal process/young barley leaves and urea or citric acid	Pseudorabies virus	mRNA expression level of IFN-α, IFN-β, and ISGs	Liu et al., 2017
Quantum dots 92.2 nm)	Microwave Synthesis/aqueous dispersed CdTe/CdS/ZnS	Human herpes simplex virus type 1	Viral replication	Hu et al., 2016
Boronic acid functionalized carbon dots	Pyrolysis/citric acid	Human immunodeficiency virus 1	Entry step	Fahmi et al., 2016
Polyamine-modified Carbon quantum dots	Pyrolysis/ spermidine powder	White spot syndrome virus	-	Jian et al., 2017; Huang et al., 2020

Polyamine-modified carbon quantum dots were proved to inhibit White spot syndrome virus infection by attaching to the viral envelope in a dose-dependant manner (Huang et al., 2020). Carbon dots were synthesized by direct pyrolysis by heating of spermidine powder (Jian et al., 2017). This virus causes white spot syndrome in cultured shrimps which has led to high mortality rates in culture shrimps around the globe. The viral inhibitory effect of this polyamine capped carbon dots was confirmed through *in vivo* experiments (Huang et al., 2020).

The above mentioned are the published researches which proved the excellent ability of these carbon derived nano-substances as antiviral agents. Works mentioning the antiviral activity of simple carbon dots as well as surface functionalized carbon dots were discussed. In most of the research mentioned here the antiviral activity is by one or more mechanism which affect the life cycle of virus. From these results we can understand that carbon dots derived from natural antiviral agents like curcumin has excellent ability against corona virus. It should be noted that surface modification or functionalization also improved the antiviral activity of carbon dots.

### Biosensing

There is a high demand for sensitive, selective, and affordable biosensors for detecting viruses in this pandemic situation. Carbon dots have been investigated in viral as well as bacterial sensing by many researchers. Environmental and biosafety make carbon dots to dominate in the diagnostic and monitoring field. Changes in fluorescence property make carbon dots to act as sensors for biological as well as non-biological entities (Jaleel and Pramod, 2018). Resonance energy transfer, inner filter property, electron transfer, photo-induced charge transfer are the main mechanism which leads to changes in fluorescence property which is required for sensing applications (Sun and Lei, 2017).

Ultrasensitive biosensor developed using carbon dots and gold nanoparticles based on the principle of fluorescence resonance energy transfer, proved to be effective for the detection of HIV DNA. The research showed promising results for real sample analysis (Qaddare and Salimi, 2017). So this method can be explored for the detection of viral RNAs also.

**Figure 3:**
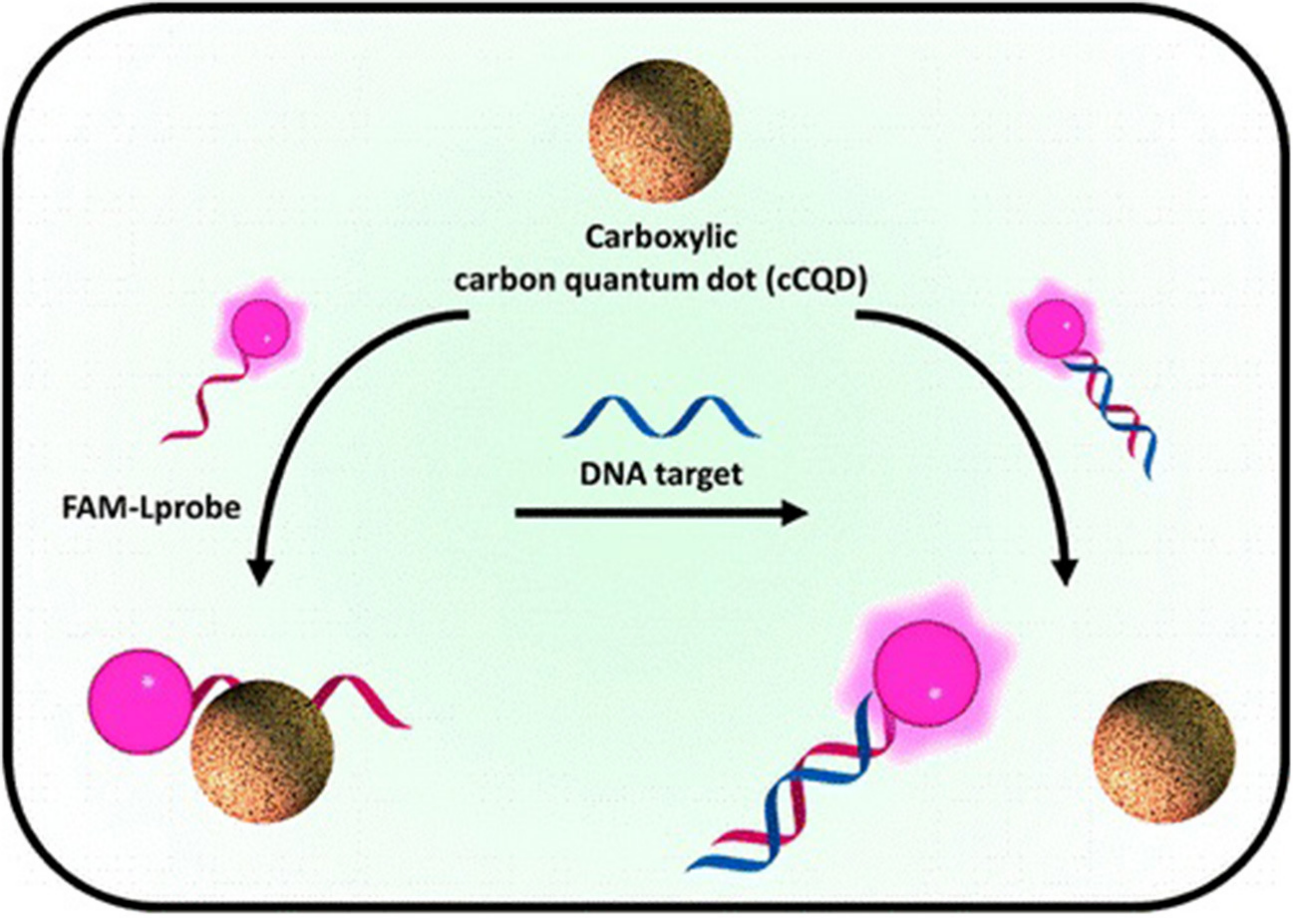
Fluorescent detection of DNA by Carboxylic acid quantum dots. Reprinted with permission from Loo et al. (2016). © (2016) American Chemical Society.

Carboxylic carbon quantum dots (citric acid and malic acid carbon dots) are useful for sensing of nucleic acid. The sensing is based on the principle that, the difference in the tendency of adsorption to the surface of carbon dots by single-stranded and double-stranded DNA (Figure 3). It was proved that citric acid and malic acid carbon dots can perform an advanced range of detection of at least 3 orders of magnitude (Loo et al., 2016). Similar way viral RNA detection probes can also be anticipated in the future. For detecting HIV DNA in biological samples a sensor containing fluorescent carbon dots and cadmium telluride quantum coated with 3-mercapropionic acid can be employed effectively. This probe allows the ratiometric determination of double-stranded DNA with a quantification limit of 1.0 nM. Also, no significant interference with biomolecules like amino acids, nucleotides, etc. was observed (Liang et al., 2017). Similarly a ratiometric nanosensor for selective recognition of DNA using fluorescent carbon dots and a fluorescent dye, ethidium bromide is also reported (Huang et al., 2015).

BAI et al., exploited carbon dots for the detection of DNA, for the first time in literature. It was observed that methylene blue can cause an excellent quenching of fluorescence of carbon dots. But the addition of ct-DNA restored the fluorescence of carbon dots since DNA can bind with methylene blue and removed it from the carbon dots. This system can detect ct-DNA with a quantification limit of 1.0 × 10^−6^ mol/L (Bai et al., 2011). Polyethylene glycol capped carbon dots also can effectively detect double-stranded DNA (Milosavljevic et al., 2015).

Carbon dots from sources such as *Saccharum officinarum* were also used for cell imaging in yeast and bacteria (Mehta et al., 2014). Carbon dots synthesized from rice straw can bind with bacterial membranes and facilitate their imaging and counting with the help of a fluorescent microscope (Mandal and Parvin, 2011). A simple as well as sensitive technique for the detection of H5N1 DNA using quantum dots and carbon nanotubes is also reported. This technique is also based on fluorescence resonance energy transfer from carbon dots to nanotubes and proved to be an easy and effective method for the quantitative detection of viral nucleic acid (Tian et al., 2012).

## Conclusion and Future Perspectives

In this current review, we have addressed all the published research as well as review articles on antiviral carbon dots along with a brief description of the synthesis and its antiviral mechanism of action. Therefore, carbon dots have proved promising application against different types of corona viruses. Still, more focus required to be given in exploring carbon dot-based antiviral agents for treating SARS-CoV, MERS-CoV, and SARS-CoV-2 viral infections. Carbon dots are extensively researched in biomedicine other than therapy like biosensing, bioimaging, etc. Surface functionalization and low toxicity makes carbon dots the most superior among other nanoparticulate therapeutic delivery systems. These functionalized carbon dots can stay as a new stage for the production of biosafe nanotherapeutics for treating viral infections in the near future. Among the reviewed researches carbon dots derived from herbal sources like curcumin, glycyrrhizin, etc. was found to be more promising because of their biocompatibility, lower toxicity, and strong *in vitro* as well as *in vivo* antiviral activity.

Apart from this, carbon dots could probably be exploited in many other ways. As there is no specific vaccine against SARS-CoV-2, cleaning of contaminated surfaces and handwashing are highly needed to prevent the spread of disease. Therefore, incorporation of carbon dots to sanitizing solutions, handwashing soaps, cleaning detergents, etc. will be useful. Carbon dot incorporated masks and air filters also seem to be promising.

What we suggest from these studies is that a bottom up approach with natural antiviral agent as a precursor for the production of carbon dots will be a promising option. Additional surface passivation/functionalization can also be considered for enhancing the efficiency. Carbon dots with positive charge and more hydrophilic groups on surface will be an add-on for a superior anti-viral action.

Regardless of the encouraging results, still much more research is needed to address some issues to make the dream come true. Firstly, the exact antiviral mechanism of these carbon dots is still not much explored and most of the literature reported an early stage inhibition except few. Secondly, the *in vivo* efficacy studies are not much detailed in any of the reported researches and it's difficult to make exact animal models for viral diseases. The mutation capability of the virus is the last but not the least issue. Still, we can expect a bright future for carbon dots in antiviral therapy especially in this urgent situation of COVID-19.

## Author Contributions

SK: conceptualization, writing—original draft, and funding acquisition. HA and NA: supervision, writing—review, and editing. SB-E, SM, AN, and PD: writing—review and editing. All authors contributed to the article and approved the submitted version.

## Conflict of Interest

The authors declare that the research was conducted in the absence of any commercial or financial relationships that could be construed as a potential conflict of interest.
